# 

**Published:** 1890-03

**Authors:** 


					﻿OLD SERIES
Vou xxy
NETT-miiS'
t? 1855 O

lEDIGAL^UR®
40URNAk



W. F. WESTMORELAND, M.D.
H. V. M. MILLER, M.D., LL.D.
VIRGIL 0. HARDON, M.D.
F. W. McRAE, M.D,, M’n’g’ng Ed.
MUSHED BY JAMES P.HARRISON&CQS
feP ___________ Atlanta >ga>
SUBSCRIPTIONS S2.BO A YEAR
				

